# Perceptions and predictors of organizational justice among healthcare professionals in academic hospitals in South-Eastern Nigeria

**DOI:** 10.1186/s12913-020-05187-5

**Published:** 2020-04-15

**Authors:** Nwanneka Chidinma Ghasi, Daniel Chukwuemeka Ogbuabor, Vincent Aghaegbunam Onodugo

**Affiliations:** 1 Department of Management, Faculty of Business Administration, University of Nigeria Enugu Campus, Enugu, Enugu State Nigeria; 2 Department of Health Administration and Management, Faculty of Health Sciences and Technology, College of Medicine, University of Nigeria Enugu Campus, Enugu, Enugu State Nigeria

**Keywords:** Organizational justice, Health professionals, teaching hospitals, Nigeria

## Abstract

**Background:**

Research on organizational justice in hospitals in African countries are limited despite being important for workforce performance and hospital operational efficiency. This paper investigated perceptions and predictors of organizational justice among health professionals in academic hospitals in South-east Nigeria.

**Methods:**

The study was conducted in two teaching hospitals in Enugu State, South-east Nigeria using mixed-methods design. Randomly sampled 360 health professionals (doctors = 105, nurses = 200 and allied health professionals, AHPs = 55) completed an organizational justice scale. Additionally, semi-structured, in-depth interview with purposively selected 18 health professionals were conducted. Univariate and bivariate statistics and multivariable linear regression were used to analyze quantitative data. Statistical significance was set at alpha 0.05 level. Qualitative data were analyzed thematically using NVivo 11 software.

**Results:**

The findings revealed moderate to high perception of different dimensions of organizational justice. Doctors showed the highest perception, whereas AHPs had the least perception. Among doctors, age and education predicted distributive justice (adjusted R^2^ = 22%); hospital ownership and education predicted procedural justice (adjusted R^2^ = 17%); and hospital ownership predicted interactional justice (adjusted R^2^ = 42%). Among nurses, age, gender and marital status predicted distributive justice (adjusted R^2^ = 41%); hospital ownership, age and gender predicted procedural justice (adjusted R^2^ = 28%); and hospital ownership, age, marital status and tenure predicted interactional justice (R^2^ = 35%). Among AHPs, marital status predicted distributive justice (adjusted R^2^ = 5%), while hospital ownership and tenure predicted interactional justice (adjusted R^2^ = 15%). Qualitative findings indicate that nurses and AHPs perceive as unfair, differences in pay, access to hospital resources, training, work schedule, participation in decision-making and enforcement of policies between doctors and other health professionals due to medical dominance. Overall, supervisors have a culture of limited information sharing with, and disrespectful treatment of, their junior colleagues.

**Conclusion:**

Perceptions of organizational justice range from moderate to high and predictors vary among different healthcare professionals. Addressing specific socio-demographic factors that significantly influenced perceptions of organizational justice among different categories of health professionals and departure from physician-centered culture would improve perceptions of organizational justice among health professionals in Nigeria and similar settings.

## Background

Skilled and motivated health workforce is an essential input to strengthen hospitals in low and middle-income countries (LMICs) [1]. Nonetheless, perception of unfair treatment of health workers reduce health workforce performance and hospital operational efficiency [1, 2]. Health workers have views about and expect fairness in the distribution of organizational resources and opportunities, wages, decision-making processes, interpersonal behaviors and provision of information within their work environment [3]. This perception of fairness or unfairness in resource allocation, decision-making and interpersonal interaction refers to organizational justice [3]. Health workers care about justice because fair actions and processes make them feel valued and motivated to perform, thus making organizational justice imperative in improving operational efficiency of hospitals [1].

Organizational justice (OJ) has been conceptualized in three dimensions: distributive justice, procedural justice and interactional justice. Distributive Justice refers to perceived fairness of how outcomes and resources are distributed among employees in organizations [4, 5]. Employees compare their outcomes such as pay, promotion and access to resources and inputs with their peers within and outside their organizations. A positive perception of distributive justice improves organizational attachment, identification and involvement [3, 4]. In contrast, distrust, disputes, disrespect and demotivation of employees occur when benefits are assigned in unfair manner [6].

Procedural justice refers to perception of fairness in the decision-making process, including motives, methods, mechanism and processes used in determining outcomes [5], and comprises: voice and process control perspectives [5, 6]. Voice involves opportunity to be heard and taken into consideration, while process control entails opportunity to influence information used in decision-making. Organizations should tolerate opinion of employees; make decisions based on consistent approach and correct information; exhibit impartiality, avoid favoritism and remain ethical; provide effective feedback; and explain decisions to employees [5]. When decision-making is perceived as fair, performance improves due to increased job involvement, organizational commitment, trust and cooperation among employees [2].

Interactional justice refers to employee perceptions of fairness of interpersonal treatment they are subjected to during decision-making procedures and comprises two dimensions: interpersonal and informational justice [7]. Interpersonal justice entails how supervisors treat co-workers with respect and dignity. Informational justice implies how supervisors share information with their subordinates relating to their tasks. Derogatory judgements, deceptions, abusive actions, public criticism and coercion result in decreased perception of interactional justice [7].

Findings on organizational justice vary in different settings. Organizational justice was found to be low [6, 8, 9], moderate [10, 11] and high [12]. Distributive justice was found to be low [6, 8, 9], moderate [10] and high [12]. Procedural justice was found to be low [6, 9] and moderate [8, 10, 12]. Interactional justice was low [6] moderate [8–10] and high [12]. Doctors had significantly high perception of organizational justice compared to moderate perception among other health professionals [11]. Whereas distributive justice and procedural justice varied significantly between doctors and other health professionals, there was no significant difference in their interactional justice [11].

Perception of unfairness in salaries decreased perception of distributive justice among healthcare professionals [3, 11, 13–20]. In Tanzania, the experience of being by-passed by colleagues with shorter working experience and longer formal training was most dissatisfying for health workers with longer working experience [14]. In Malawi, lack of clear criteria for promotion after upgrading their qualification decreased distributive justice among nursing staff and clinical officers [19]. Access to training differed among different categories of nurses in Tanzania and Malawi [14, 20]. Doctors, unlike other health professionals, have access to residency training [17]. Iranian nurses have less access to hospital resources than doctors [18, 21]. Nurses lack control over their practice setting, get assigned physician duties and experience unwarranted interference by doctors in Iran [18, 21]. Also, public hospitals were found to have physician-centered culture where doctors are seen as superior to other health professionals [15–18, 21, 22].

Studies indicate that doctors participated more in decision-making than nurses and other health professionals [3, 9, 14–16, 21–23]. Australian nurses perceive organizational policies and procedures as being fair [4]. In contrast, junior nurses in Turkey perceived unfairness in their performance assessment while hospital management were more lenient to doctors than nurses in disciplinary procedures [9, 18]. Doctors lacked respect for other health professional [16]. Health managers tend to be authoritarian, unsupportive, overuse behavioral control, lack respect for junior staff and provide negative feedbacks during supervision [2, 4, 18–21, 23–27].

In Nigeria, evidence of organizational injustice, deriving from studies on industrial actions, inter-professional conflict, and health workforce governance highlight the huge organizational challenges in managing and motivating healthcare professionals for effective performance in Nigeria especially importance of rewards, professional dominance and work climate [13, 15–17, 28]. Although there are growing concerns about whether rewards and hospital resources are allocated to healthcare professionals equitably and whether such allocation decisions are made according to fair methods and guidelines, studies on perceptions of organizational justice among health workers in Nigeria are scarce [11]. Since evidence indicate that employees’ perception of fairness is positively related to job satisfaction, perceived organizational support, leader–member exchange, task performance, work engagement, organizational commitment and organizational citizenship behaviour; and negatively related to job burnout, turnover intentions and counterproductive work behaviour [29–31]; it is imperative to provide context-specific evidence of healthcare professionals’ perceptions of organisational justice in Nigeria. Songstad et al. argue that, to improve motivation for work and work performance, an approach which takes issues at microlevel, the health facility, should be the starting point of efforts to identify factors behind perceptions of unfairness in health workers’ working conditions [14]. The purpose of this study, therefore, was to investigate the perceptions and predictors of organizational justice among different categories of health professionals in public tertiary hospitals. Such information, which enhances understanding of work climate of healthcare professionals, will be more useful to hospital managers and decision makers in Nigeria and similar settings in revising human resources policies and promoting employee-centred practices to improve operational efficiency of public hospitals.

## Materials and methods

### Study setting

The study took place in two university teaching hospitals in Enugu State, South-east Nigeria: Enugu State University Teaching Hospital (ESUTH) and University of Nigeria Teaching Hospital (UNTH) with comparable clinical departments and cadres of health professionals. ESUTH has 839 health professionals consisting of 257 doctors, 455 nurses/midwives, and 127 allied health professionals (AHPs). UNTH has 1399 health professionals comprising 397 doctors, 800 nurse/midwives, and 202 AHPs. In this study, AHPs comprises pharmacists, medical laboratory scientists, physiotherapists optometrists and radiographers. Both hospitals provide apex health services to about 5 million people in Enugu State.

### Research design

The study adopted a mixed methods design. The quantitative component involved cross-sectional questionnaire survey, while the qualitative part was based on semi-structured, in-depth interview (IDI) of eligible healthcare professionals.

### Sampling and sample size

#### Quantitative

All 2238 health professionals directly involved in clinical care of patients in the two teaching hospitals constituted the study population. The sample size of 352 participants was calculated based on single population proportion formula in a finite population [32]. We assumed that 50% of the population will have unfair perception of their workplace treatment, a tolerable error of 5, 95% confidence level and 10% non-response rate. Nonetheless, we increased the sample to 360 health professionals. Proportionate stratified sampling technique was used to allocate 37.5 and 62.5% of the sample size ESUTH and UNTH respectively [33]. Healthcare professionals were included in the study if they were directly involved in clinical care of patients in the hospitals; have been employed for at least 1 year preceding the survey; were available during the period of the study; and willing to participate in the study. We excluded healthcare professionals who were not involved in clinical care of patients such as health information, environmental and public health professionals; have been tenured for < 1 year; and those on any form of leave. In each hospital, samples were allocated to doctors, nurses and AHPs proportionate to their size. Health professionals were randomly selected from each stratum.

#### Qualitative

We purposively selected 18 health professionals from among survey respondents, 9 per teaching hospital (3 doctors, 3 nurses and 3 AHPs). To ensure maximum variation, each group of health professionals included both male and female professionals, those with managerial role, leaders of their professional association, and/or frontline service providers. All participants had worked in their hospitals for at least 5 years.

### Data collection tool and data collection

#### Quantitative

A pre-tested validated questionnaire was used to collect data from health professionals from January to March 2018. The questionnaire was self-administered and included sections socio-demographic characteristics of respondents and organizational justice. The organizational justice scale was adapted from a validated questionnaire used in a previous study [34], and consisted of 20-item (distributive justice =5; procedural justice = 6; and interactional justice = 9) scored on a 5-point Likert scale (strongly disagree = 1, disagree = 2, undecided = 3, agree = 4, strongly agree = 5). We conducted exploratory factor analysis using principal component analysis and varimax rotation to validate the organizational justice scale. The Kaiser-Meyer-Olkin (KMO) measure of sampling adequacy was 0.877 (X^2^ = 5281.68, ρ = 0.000). Since a KMO of 0.5 is considered adequate, the data was deemed suitable for factor analysis [35]. All 20 items of the scale have communalities ≥0.5 before rotation and were judged adequate [36]. After varimax rotation with Kaiser normalization, all 20 items also loaded ≥0.5, which were regard as adequate item loading, and were retained in the scale [36]. The reliability coefficient of the organizational justice scale among our sample was 0.915. The reliability coefficients of the sub-scales were 0.804, 0.749 and 0.872 for distributive, procedural and interactional justice correspondingly. All 360 questionnaires distributed were fully completed and used in this study.

#### Qualitative

We conducted 18 semi-structured, in-depth interviews with healthcare professionals using an interview guide we developed based on dimensions of organizational justice (Additional file 1). The interview guide covered perceptions of salaries and rewards, promotion, equal access to training, work schedule, access to hospital resources, participatory decision-making, employee voice, appeal process, respectful treatment of workers, concern for the rights and personal needs of health workers and adequate explanation for job decisions. Participants were identified using leaders of respective health professions in each hospital as gatekeepers. Written, informed consent was obtained from each participant for participation and audiotaping of the interview. The interviews, which were conducted by the first and second authors, were held in English language, at a venue and a time chosen in consultation with the participants and each lasted about 60 min.

### Data analysis

#### Quantitative

The sample was analyzed as a whole and by professional groups using SPSS (version 20, IBM, New York, USA). The data were checked by tests for skewness and kurtosis to ensure that the assumptions of normality were met [37]. Both skewness and kurtosis lie between − 1 but and + 1, indicating normal distribution. We combined the responses from several related questions in each dimension of organizational justice into single composite scores with interval-level properties [38]. These assumptions notwithstanding, parametric tests can be used to analyse Likert scale responses, even when statistical assumptions such as a normal distribution of data, are violated to extreme degree [39, 40]. Univariate analyses of organizational justice and its dimensions were done using descriptive statistics (mean and standard deviation, frequencies and percentages). The mean scores were reported on a scale of 1–5, with higher values corresponding to higher perception of organizational justice. Bivariate analysis of association of organizational justice and its dimensions with socio-demographic factors (SDFs) of respondents was done using t- tests and analysis of variance (ANOVA). Predictors of organizational justice of health professionals were established using multivariable linear regression. Statistical significance was set at alpha 0.05 level.

#### Qualitative

The interviews were transcribed verbatim and analyzed thematically using NVivo software (version 11, QSR International Pty Ltd., Victoria, Australia). The main themes were deduced from dimensions of OJ. The sub-themes were generated, inductively, by reading the transcripts and reflected organizational factors that characterize each dimension of organizational justice. Two persons coded the transcripts and resolved inter-coder differences by consensus. To ensure rigor, we sent back the transcripts to participants for validation and used excerpts to illustrate the findings.

### Triangulation

Data from the quantitative and qualitative components, although analyzed separately, were triangulated at interpretive level to enrich the findings from both sources.

### Ethical consideration

Ethical clearance was obtained from the Health Research Ethics Committee of the University of Nigeria Teaching Hospital, Enugu, Nigeria.

## Results

### Quantitative findings

The socio-demographic characteristics of respondents are shown in Table 1. Most doctors and AHPs were males, while nurses were mostly females. Most health professionals were less than 40 years, married, held a bachelor’s degree or less and have worked for less than 10 years.

**Table 1:** Socio-demographic characteristics of respondents

SDF	Overall (***N*** = 360)	Doctors (***N*** = 105)	Nurses (***N*** = 200)	AHPs (***N*** = 55)
	**n (%)**	**n (%)**	**n (%)**	**n (%)**
**Hospital**				
State hospital	135 (37.5)	41 (39)	73 (36.5)	21 (38.2)
Federal hospital	225 (62.5)	64 (61)	127 (63.5)	34 (61.8)
**Gender**				
Male	149 (41.4)	96 (91.4)	(4.0)	45 (81.8)
Female	211 (58.6)	9 (8.6)	192 (96.0)	10 (18.2)
**Age**				
20–29	67 (18.6)	27 (25.7)	36 (18.0)	4 (7.3)
30–39	187 (51.9)	43 (41.0)	112 (56)	32 (58.2)
40–49	83 (23.1)	30 (28.6)	38 (19.0)	15 (27.3)
50 and above	23 (6.4)	5 (4.8)	14 (7.0)	4 (7.3)
**Marital status**				
Never Married	93 (25.8)	34 (32.4)	41 (20.5)	18 (32.7)
Married	267 (74.2)	71 (67.6)	159 (79.5)	37 (67.3)
**Education**				
≤ bachelor	308 (85.6)	82 (78.1)	190 (95.0)	36 (65.5)
≥ Masters*	52 (14.4)	23 (21.9)	10 (5.0)	19 (34.5)
**Tenure**				
< 10 yrs	274 (76.1)	99 (94.3)	134 (67.0)	41 (74.5)
≥ 10 years	70 (19.5)	6 (5.7)	66 (33.0)	14 (25.5)

*Includes doctors with fellowship

The mean organizational justice score for the entire sample was 2.86 (0.73). Interactional justice had the highest, while distributive justice had the lowest score. Significant differences in mean scores for overall organizational justice, distributive justice, procedural justice and interactional justice were found among health professional subgroups (Table 2).

**Table 2:** Mean scores (standard deviation)^1^ of organizational justice and its dimensions for the entire sample and by health profession

	Overall (*N* = 360)	Doctors (*N* = 105)	Nurses (*N* = 200)	AHPs (*N* = 55)
Distributive justice	2.67 (0.90)	3.25 (0.78)	2.44 (0.86)	2.42 (0.71)
Procedural justice	2.75 (0.89)	3.25 (0.46)	2.58 (0.96)	2.42 (0.85)
Interactional justice	3.12 (0.89)	3.77 (0.44)	2.93 (0.88)	2.58 (0.88)
Overall organizational justice	2.86 (0.73)	3.35 (0.62)	2.71 (0.71)	2.47 (0.54)

^1^ Reported on a 1–5 scale with higher values corresponding to higher perception of justice

The mean scores for distributive justice by SDFs are shown in Table 3. Perception of distributive justice differed significantly across age groups and education among doctors. Among nurses, the mean scores differed significantly by all SDFs except hospital ownership. Never married respondents had significantly higher scores than their married colleagues among AHPs.

**Table 3:** Mean scores (standard deviation)^1^ by socio-demographic factors for distributive justice

SDF		Overall (*N* = 360)	Doctors (*n* = 105)	Nurses (*n* = 200)	AHPs (*n* = 55)
Hospital ownership	State	2.61 (0.82)	3.34 (0.69)	2.29 (0.70)	2.33 (0.48)
	Federal	2.71 (0.94)	3.19 (0.83)	2.53 (0.93)	2.47 (0.83)
	**Sig.**^**2**^	0.346	0.327	0.057	0.493
Gender	Male	2.89 (0.90)	3.2 (0.78)	1.50 (0.53)	2.44 (0.73)
	Female	2.52 (0.86)	3.56 (0.53)	2.48 (0.85)	2.30 (0.68)
	**Sig**^**2**^**.**	0.000*	0.218	0.001*	0.567
Age					
	20–29	3.36 (0.62)	3.11 (0.64)	3.61 (0.49)	2.75 (0.50)
	30–39	2.50 (0.79)	3.05 (0.79)	2.30 (0.71)	2.47 (0.76)
	40–49	2.61 (0.10)	3.60 (0.77)	1.97 (0.59)	2.27 (0.59)
	50 and above	2.26 (1.01)	3.60 (0.89)	1.79 (0.58)	2.25 (0.96)
	**Sig**^**3**^	0.000*	0.011*	0.000*	0.598
Marital status	Single	3.13 (0.78)	3.15 (0.66)	3.27 (0.90)	2.78 (0.65)
	Married	2.51 (0.88)	3.30 (0.84)	2.23 (0.71)	2.24 (0.68)
	**Sig**^**2**^	0.000*	0.364	0.000*	0.008*
Education	≤Bachelors	2.71 (0.92)	3.39 (0.75)	2.47 (0.87)	2.42 (0.73)
	≥Masters	2.46 (0.73)	2.74 (0.69)	1.90 (0.57)	2.42 (0.69)
	**Sig**^**2**^	0.066	0.000*	0.041*	0.983
Tenure	< 10 years	2.82 (0.90)	3.25 (0.80)	2.63 (0.89)	2.44 (0.74)
	> 10 years	2.19 (0.70)	3.17 (0.41)	2.06 (0.65)	2.36 (0.33)
	**Sig**^**2**^	0.000*	0.795	0.000*	0.714

^1^ Reported on a 1–5 scale with higher values corresponding to higher perception of justice

^2^ According to t-test

^3^ According to ANOVA

*Significant

Higher educational status a and working in state-owned teaching hospital was related to low perception of procedural justice among doctors (Table 4). All the SDFs had significant mean score differences among nurses. Significant mean score differences in perception of procedural justice were found among AHPs by age and tenure.

**Table 4:** Mean scores (standard deviation) by socio-demographic factors for procedural justice

SDF		Overall (N = 360)	Doctors (n = 105)	Nurses (*n* = 200)	AHPs (n = 55)
Ownership	State	2.84 (0.78)	3.10 (0.37)	2.86 (0.89)	2.24 (0.70)
	Federal	2.70 (0.94)	3.34 (0.48)	2.43 (0.96)	2.53 (0.93)
	**Sig.**^**2**^	0.162	0.006*	0.002*	0.222
Gender	Male	2.93	3.25	1.63 (0.74)	2.47 (0.87
	Female	2.63	3.22	2.63 (0.95)	2.20 (0.79)
	**Sig.**^**2**^	0.002*	0.862	0.004*	0.377
Age					
	20–29	3.18 (0.46)	3.37 (0.49)	3.11 (032)	2.50 (0.58)
	30–39	2.75 (0.96)	3.28 (0.45)	2.73 (1.04)	2.13 (0.75)
	40–49	2.49 (0.82)	3.10 (0.40)	1.92 (0.63)	2.73 (0.88)
	50 and above	2.43 (0.99)	3.20 (0.45)	1.86 (0.77)	3.50 (0.58)
	**Sig**^**3**^	0.000*	0.146	0.000*	0.004*
Marital status	Single	3.01 (0.59)	3.32 (0.48)	3.05 (0.31)	2.33 (0.77)
	Married	2.66 (0.95)	3.21 (0.45)	2.47 (1.03)	2.46 (0.90)
	**Sig**^**2**^	0.001*	0.239	0.000*	0.612
Education	≤Bachelors	2.79 (0.88)	3.30 (0.49)	2.63 (0.94)	2.42 (0.77)
	≥Masters	2.56 (0.90)	3.04 (0.21)	1.70 (0.95)	2.42 (1.02)
	**Sig**^**2**^	0.086	0.014*	0.003*	0.986
Tenure	< 10 years	2.86 (0.84)	3.26 (0.47)	2.76 (0.94)	2.22 (0.73)
	> 10 years	2.41 (0.94)	3.00 (0.00)	2.23 (0.91)	3.00 (0.96)
	**Sig**^**2**^	0.002*	0.171	0.000*	0.002*

^1^ Reported on a 1–5 scale with higher values corresponding to higher perception of justice

^2^ According to t-test

^3^ According to ANOVA

*Significant

The mean scores by SDFs for interactional justice are shown in Table 5. Mean scores for IJ significantly differed by hospital ownership and education among doctors. Among nurses, only education did not show significant mean score differences in interactional justice. Hospital ownership and tenure showed significant mean score differences among AHPs.

**Table 5:** Mean scores (standard deviation) by socio-demographic factors for interactional justice

SDF		Overall (N = 360)	Doctors (n = 105)	Nurses (n = 200)	AHPs (n = 55)
Hospital Ownership	State	3.18 (0.71)	3.41 (0.55)	3.12	2.90 (0.30)
	Federal	3.09 (0.98)	4.00 (0.00)	2.82	2.38 (1.05)
	**Sig**^**2**^	0.360	0.000*	0.018*	0.030*
Gender	Male	3.31 (0.89)	3.78 (0.44)	2.13 (0.35)	2.51 (0.92)
	Female	2.99 (0.87)	3.67 (0.50)	2.96 (0.88)	2.90 (0.57)
	**Sig**^**2**^	0.001*	0.462	0.008*	0.207
Age					
	20–29	3.79 (0.59)	3.81 (0.40)	3.83 (0.68)	3.25 (0.50)
	30–39	3.02 (0.85)	3.84 (0.37)	2.89 (0.78)	2.34 (0.75)
	40–49	2.88 (0.88)	3.70 (0.54)	2.26 (0.45)	2.80 (0.94)
	50 and above	2.91 (1.08)	3.40 (0.55)	2.71 (1.14)	3.00 (1.41)
	**Sig**^**3**^	0.000*	0.139	0.000*	.084
Marital status	Single	3.53 (0.87)	3.82 (0.39)	3.73 (0.84)	2.50 (0.86)
	Married	2.98 (0.86)	3.75 (0.47)	2.72 (0.77)	2.62 (0.89)
	**Sig**^**2**^	0.000*	0.408	0.000*	.633
Education	≤Bachelors	3.09 (0.89)	3.71 (0.48)	2.95 (0.89)	2.44 (0.88)
	≥Masters	3.31 (0.85)	4.00 (0.00)	2.60 (0.70)	2.84 (0.83)
	**Sig**^**2**^	0.086	0.005*	0.226	0.110
Tenure	< 10 years	3.24 (0.86)	3.76 (0.45)	3.10 (0.87)	2.41 (0.77)
	> 10 years	2.76 (0.89)	4.00 (0.00)	2.58 (0.81)	3.07 (0.10)
	**Sig**^**2**^	0.000*	0.171	0.000*	0.014*

^1^ Reported on a 1–5 scale with higher values corresponding to higher perception of justice

^2^ According to t-test

^3^ According to ANOVA

*Significant

Table 6 shows how specific SDFs predicted the three dimensions of organizational justice among different categories of health professionals.

**Table 6:** Multivariate analysis of organizational justice dimensions by health professions

	B Coefficient (***p*** value)*								
**Model**	**Distributive justice**			**Procedural justice**			**Interactional justice**		
	**Doctors**	**Nurses**	**AHPs**	**Doctors**	**Nurses**	**AHPs**	**Doctors**	**Nurses**	**AHPs**
(Constant)	3.27	2.12	3.86	3.14	3.76	1.30	2.358	4.640	2.170
	(0.000)	(0.001)	(0.000)	(0.000)	(0.000)	(0.158)	(0.000)	(0.000)	(0.024)
Hospital ownership				0.39	−0.59		.586	−.479	−.525
				(0.000)	(0.000)		(0.000)	(0.000)	(0.045)
Age	0.43	− 0.53			−0.44			−.348	
	(0.000)	(0.000)			(0.000)			(0.000)	
Gender		0.86			0.71				
		(0.001)			(0.019)				
Marital status		−0.47	−0.59					−.640	
		(0.001)	(0.027)					(0.000)	
Education	−0.88			− 0.39					
	(0.000)			(0.001)					
Tenure								−.268	.667
								(0.046)	(0.048)
Adjusted R^2^ =	0.222	0.411	0.048	0.168	0.279	0.148	0.423	0.353	0.147

*Beta coefficient (*p* value) of only significant results are presented

### Qualitative findings

#### Distributive justice

Unfair pay, leadership and recognition, access to hospital resources, promotional opportunities, training opportunities and work schedule emerged as key themes in distributive justice.

#### Unfair pay

Nurses and AHPs stated that pay disparity between doctors and non-doctors is high. In contrast, doctors argued that *‘relativity (pay differences) is a global norm and should apply to Nigeria’* (Doctor 4). Whereas non-doctors stated that *‘the consolidated medical salary scale has been adjusted thrice’* (Nurse 3); doctors claimed that enhancement of the doctors’ pay scheme was to restore relativity across health professionals, which *‘has been eroding over the past 20 years’* (Doctor 2).

In the state teaching hospital, Nurses and AHPs are not paid with nationally approved salary scale *‘6 to 7 years after doctors received the new salary’* (Nurse 5). Also, the salaries of doctors are *‘about half of their contemporaries in federal-owned hospitals’* (Doctor 5). Furthermore, *‘the doctors in the State Ministry of Health receive higher pay than those in the teaching hospital’* (Doctor 5).

##### Leadership and recognition

Nurses and AHPs stated that doctors are more recognized in hospitals and head tertiary hospitals in Nigeria despite that *‘the act establishing teaching hospitals did not prescribe that only doctors should head hospitals’* (AHP 6). Doctors argued that the doctor should lead the health team and *‘has to oversee what is done for the patient’* (Doctor 1).

#### Access to hospital resources

AHPs and nurses indicated that doctors have more access to hospital resources than other health professionals. Non-doctors argued that *‘doctors have stronger power to negotiate with hospital management to get what they want’* (AHP 5) and that *‘the management singles out a particular profession to favor them in everything’* (Nurse 3). Doctors claimed that *‘doctors have fewer office space allocation than other health professionals’* (Doctor 3). Few nurses observed that AHPs *‘have more access to hospital resources than nurses’* (Nurse 5).

#### Promotional opportunities

All health professional sub-groups agreed that promotion is often delayed, notional and without financial benefits. However, nurses and AHPs do not get promoted to the rank of director because *‘the law establishing teaching hospitals recognized only 2 directors’* (AHP 4). Nurses also stated that *‘nurses use their off-duty and shift time to go through school, but hospital management would not endorse their certificate’* for promotion (Nurse 5), unlike resident doctors. Doctors explained that although, management cannot stop nurses from unapproved in-service training, it can stop them *‘from benefiting from that degree’* (Doctor 6).

#### Training opportunities

AHPs and nurses indicated that doctors have more access to training. As explained by nurses, *‘a nurse who wants to acquire further education and training does that at his own time with his own money’* (Nurse 3). Yet, *‘resident doctors are on training and receive their full salaries’* (nurse 5). Doctors argued that *‘teaching hospitals are primarily a place where doctors are trained’* (Doctor 4) *‘to improve their clinical competencies’* (Doctor 5), in contrast to other health professionals whose academic qualifications are not relevant to patient care.

AHPs and nurses indicated that *‘when doctors go for their primaries, they are reimbursed, but when others attend workshops, management will tell them that there is no money’* (AHP 2). However, doctors indicated that refunds for update courses, examinations and conferences are often delayed, *‘and when they pay, they may just pay a part’* (Doctor 4).

#### Work schedule

AHPs said that doctors take precedence over other health workers when work schedules conflicted: *‘one will be in the ward seeing a patient, the medical team comes and says my chief is here, we want to use the folder. What is this?’* (AHP 2). Nurses indicated that *‘doctors virtually leave their job descriptions to nurses’* (nurse 5). Yet, nurses are not allowed by doctors to do certain clinical procedures such as *‘administering intravenous drugs’* (Nurse 3). Doctors insisted that patient care rests on doctors, who should ‘*call in anyone whose expertise is needed to give patients the best healthcare available’* (Doctor 1).

### Procedural justice

Consultation and representation, and appealing management decisions emerged as two themes in procedural justice.

#### Consultation and representation

All participants agreed that *‘managers of hospitals take most decisions without involving workers’* (AHP 6) and *‘such decisions are cascaded down to workers no matter what you think’* (Doctor 4). However, AHPs and nurses indicated that other health professionals, unlike doctors, lack power in the decision-making process in hospitals. One AHP opined that: *‘granted that doctors become chief medical directors but when it comes to choosing one, other health professionals should be involved’* (AHP 4). In contrast, doctors stated that the medical advisory committee provided a *‘platform for every profession, represented by heads of various departments, to be involved in the decision-making process’* (Doctor 2). Nurses said that *‘occasionally, they (hospital management) involve the head of nursing service but she wouldn’t come out openly to tell them what is needed’* (Nurse 4). Doctors explained that *‘nurses are mostly female, and their voice is not loud’* (Doctor 5).

#### Appeal management decisions

All categories of health professionals can appeal management decisions through formal reports to hospital management, but most times, such appeals have been ineffective. Labor unions represent health professionals in such appeals *‘because the civil service rule does not permit individual workers to raise issues against management’* (AHP 4). However, AHPs and nurses argued that *‘when doctors appeal unfavorable decisions, management more readily listens to them’* (AHP 6). Doctors explained that *‘those decisions would have been made in the best interest of the patient’* and *‘because of the level of people that lead the doctors … they find it much easier to interact with management*’ (Doctor 5).

### Interactional justice

Enforcement of policies and procedures, information sharing, and dignity and respect emerged as key themes in interactional justice.

#### Enforcement of policies and procedures

Doctors claimed that *‘when it comes to disciplinary measures, management tend to be more ruthless with doctors than other health professionals. Hardly would you hear that a non-doctor is suspended*’ (Doctor 4). AHPs and nurses stated that hospital management is more lenient with doctors in complying with policies and rules. For instance, *‘when others embarked on industrial action, their salaries were withheld, but when doctors went on strike for 3 months, they received their salaries’* (Nurse 3).

#### Information sharing

Among AHPs and nurses, information-sharing is limited because *‘the senior ones are not available most times’* (AHP 2). A supervisor would rather *‘keep information to oneself than pass it to the supervisee. Yet, when one makes a mistake, one will go in for it’* (Nurse 4). Doctors indicated that there is transition from an approach whereby the senior doctors handed over instructions to junior doctors without asking for their input to a more supportive supervisory approach. However, one doctor argued that *‘if consultants will be at work for one third of the time, the rate of mortality will drop by more than half’* (Doctor 6).

#### Dignity and respect

AHPs stated that supervisors treat their junior colleagues respectfully, but doctors and nurses observed that *“the supervision of medical laboratory scientist is very porous”* (Nurse 5). Supervision among doctors has conventionally been fault-finding in which senior doctors made derogatory statements about junior doctors before patients and other health professionals such as *‘Aturu (sheep) or goat*’ (Doctor 1). A nurse observed that *‘if you see a senior registrar insulting a junior registrar, you would think that the junior doctor did not go to school’* (Nurse 4).

Furthermore, senior nurses leave duties for their younger colleagues; yet make derogatory judgements about them: *“I have had a fair share of being insulted and humiliated by my senior … They blame, judge you, they make people to feel less important”* (Nurse 4). Whereas doctors perceived that *‘opinions of younger nurses do not count’* (Doctor 4), AHPs explained that gender norms influenced how senior nurses treated their junior colleagues: ‘it is a profession dominated by women and they have their idiosyncrasies … if they like you, they like you; if they don’t like you, you can hardly please them’ (AHP 2).

## Discussion

This study revealed that health professionals had moderate perception of distributive justice. Our findings are similar to evidence from a previous study [10], but contradict low [6, 8, 9], and high [12], distributive justice among health workers in other studies. The moderate level of distributive justice might be due to perception by all health professionals that salaries, promotional opportunities and hospital resources are generally insufficient. Inadequate pay and hospital resources have been reasons for industrial actions in Nigeria [13, 15–17, 28]. However, our findings that nurses and AHPs had significantly lower perception of distributive justice than doctors validate findings of a previous Nigerian study [11]. Three factors could explain these findings. First, pay disparity between doctors and non-doctors were perceived as unfair by non-doctors. Evidence show that unfairness in health sector salaries decreased perception of distributive justice among health professionals [3, 11, 13–18]. Secondly, doctors seem to have more access to leadership and managerial positions, hospital resources, training opportunity and preferences in work schedules than non-doctors in teaching hospitals. Public hospitals have also been shown to have physician-centered in previous studies [15–18, 21, 22]. Thirdly, the differences in perception of distributive justice among different categories of health professionals were mediated by specific socio-demographic factors. For instance, whereas increasing age predicted high perception of distributive justice among doctors age was inversely related to distributive justice among nurses.

Our findings on distributive justice highlight three meaningful changes in the broader context of teaching hospitals. First, although salaries of health professionals are not determined by individual hospitals, salary schemes should be based on comprehensive job evaluation to ensure equity [4–6]. Secondly, the laws establishing teaching hospitals are physician-centered in terms of hospital leadership. The national and state parliaments should amend these laws to remove any ambiguities that suggest that a profession is favored. Relatedly, hospital managers need to depart from physician-centered culture and distribute hospital resources and opportunities fairly. Furthermore, the socio-demographic differences should be incorporated into human resource management strategy to improve distributive justice among health professionals in public hospitals.

This study also found moderate perception of procedural justice among health professionals, similar to evidence from previous studies [8, 10, 12], but contrasts low procedural justice in other studies [6, 9]. Participatory decision-making was generally low in teaching hospitals as formal mechanisms for consultation with health workers are lacking. Yet, consistent with findings of previous studies [3, 9, 11, 14–16, 21–23], nurses and AHPs had significantly lower perception of procedural justice than doctors in this study. This is, in part, because doctors seem to have more power than other health professionals in decision-making process and engagement with hospital management. Additionally, specific personal attributes influence perception of procedural justice among different health professional groups. For example, lower perception of procedural justice among doctors from state-owned teaching hospital might have resulted from difficulties in negotiating working conditions and labor agreements with the state government. Working in federal hospital was also predictive of low procedural justice among nurses because nurses are unable to influence decisions affecting their career progression. To improve procedural justice in teaching hospitals, hospital managers must create equal opportunities for different categories of health workers to be heard, provide feedback and explain decisions to employees [5].

Overall, this study revealed a high perception of interactional justice among health professionals as in a previous study [12], but dissimilar to findings of low [6], or moderate [8–10], interactional justice in other studies. Notwithstanding its high perception, interactional justice was limited by reduced information sharing with, and lack of respect for subordinates, by supervisors among all professional sub-groups. Our findings support the evidence that health managers tend to be unsupportive, lack respect for junior staff and provide negative feedbacks during supervision [2, 4, 18–21, 23–27]. The study further revealed that nurses and AHPs had significantly lower perception of interactional justice than doctors, which contrasts insignificant differences in interactional justice between doctors and non-doctors found in a previous Nigerian study [11]. Hospital managers in our study setting seem to be more lenient with doctors than other health professionals when enforcing policies and procedures, which is similar to findings elsewhere [9, 18]. Likewise, doctors seem to show concern about their junior colleagues more than other health professionals. Equally, some socio-demographic factors mediate differences in perception of interactional justice among health professionals. Doctors in the state-owned hospital had significantly lower interactional justice than their colleagues at the federal hospital. It is possibly that doctors in the federal hospital show more co-worker concern than their colleagues in the state hospital. It could also be that the transition from unsupportive to supportive supervision among doctors is better implemented in the federal hospital. In the federal hospital, working for more than 10 years predicted low interactional justice among nurses, whereas working for less than 10 years predicted low interactional justice among AHPs. Hospitals’ human resource policies must emphasize strict adherence to hospital policies across all health professions; information sharing with and respectful treatment of all staff; and address socio-demographic peculiarities of different health professionals.

This study provides useful insights into how health professionals in Nigerian public hospitals cognitively approach and frame organizational justice and the conditions for enactment of injustice. Nonetheless, the study could potentially be limited by social desirability associated with cross-sectional surveys, but anonymity, use of validated questionnaire and good communication between researchers and respondents greatly reduced the bias. Additionally, triangulation with qualitative findings increased the validity of our conclusions.

## Conclusion

Overall, distributive and procedural justice were moderate, whereas interactional justice was high among healthcare professionals in this study. Yet, nurses and AHPs had significantly lower perception across three dimensions of organizational justice than doctors. Specific set of socio-demographic factors significantly influenced perception of organizational justice among different categories of health professionals. Nurses and AHPs perceive as unfair, differences in pay, access to hospital resources, training, work schedule, participation in decision making and enforcement of policies between doctors and other health professionals due to medical dominance. We conclude that teaching hospitals have a need to depart from physician-centered culture and distribute hospital resources and opportunities fairly across all health professional sub-groups. Hospital managers must create equal opportunities for different categories of health workers to participate in decision-making. Strict enforcement of hospital policies across all health professions, information sharing with and respectful treatment of all health professionals are imperative. Furthermore, it is imperative to incorporate socio-demographic differences into hospitals’ human resource management strategy.

## Supplementary information

**Additional file 1.** Appendix A. In-depth interview guide for healthcare professionals. The tool was used to guide data collection from healthcare professionals. The purpose of this interview is to explore the experiences of organizational (in) justice among healthcare professionals in academic hospitals in Enugu State, South-eastern Nigeria.

## Data Availability

The datasets generated and/or analysed during the current study are not publicly available but are available from the corresponding author on reasonable request.

## Abbreviations

AHPs: Allied health professionals
BSc: Bachelors of science
CNO: Chief nursing officer
CONESS: Consolidated health salary scale
DJ: Distributive justice
ESUTH: Enugu State University Teaching Hospital
IDI: In-depth interview
IJ: Interactive justice
LMICs: Low and middle-income countries
OJ: Organisational justice
PJ: Procedural justice
SDFs: Socio-demographic factors
UNTH: University of Nigeria Teaching Hospital
