# Supplementary material for: Perceptions and predictors of organizational justice among healthcare professionals in academic hospitals in South-Eastern Nigeria
Source: BMC Health Serv Res. 2020 Apr 15;20:301. doi: 10.1186/s12913-020-05187-5 (PMC7158107; doi:10.1186/s12913-020-05187-5)
Supplement: Supplementary file 1 — Additional file 1. Appendix A. In-depth interview guide for healthcare professionals. The tool was used to guide data collection from healthcare professionals. The purpose of this interview is to explore the experiences of organizational (in) justice among healthcare professionals in academic hospitals in Enugu State, South-eastern Nigeria. [file 12913_2020_5187_MOESM1_ESM.docx]

**APPENDIX A: IN-DEPTH INTERVIEW OF HEALTH PROFESSIONALS**

The purpose of this interview is to explore the experiences of organizational (in)justice among healthcare professionals in academic hospitals in Enugu State, South-eastern Nigeria.

**Distributive justice**

1. Describe your perception of the salaries, rewards, bonuses, which your category of health professional receive. Probe for
2. fairness based on job input,
3. equal with that of other health professionals and
4. use of approved pay scheme to all health professionals.
5. In your own view, are all health Professionals based on their skills and competencies, treated fairly in terms of
6. Promotion
7. Equal access to trainings
8. Equal recognition
9. Fair work schedule
10. Equal access to hospital resources to do their work

Probe for key challenges that limit job satisfaction of health professionals

**Procedural justice (Participatory decision-making):**

1. Describe how are job decisions made and how healthcare professionals are consulted, and their concern heard before changes are implemented in your hospital? Probe for
2. Collection and use of accurate and complete information, not biased?
3. How management clarifies decisions and provides additional information when requested by all health professionals?
4. How health professionals appeal job decisions that are made by their managers/ supervisors
5. How (non) participation affects healthcare professionals’ commitment, sense of belonging and feelings to spend the rest of their career in this hospital?

**Interactional justice**

1. Describe how your hospital management enforce organisational policies and procedures. Probe for
2. Conflict resolution among different health professionals in a fair manner
3. Whether performance management policy, disciplinary policy and procedures are consistently to all health professionals
4. How unfairness in enforcement of hospital policies makes health professionals show counter-productive work behaviour
5. **Describe the relationship between** Heads of Departments/ Supervisors and their subordinates among different healthcare professionals. Probe for
6. Respectful treatment of subordinates
7. Concern for the rights and personal needs of health workers
8. Provision of clear information and justification for decisions to health professional in clear and honest manner.
9. How the relationship between your heads of departments/ supervisors affect the task performance of health professionals in your hospital.
